# An insight into the sialome of the oriental rat flea, *Xenopsylla cheopis *(Rots)

**DOI:** 10.1186/1471-2164-8-102

**Published:** 2007-04-16

**Authors:** John F Andersen, B Joseph Hinnebusch, David A Lucas, Thomas P Conrads, Timothy D Veenstra, Van M Pham, José MC Ribeiro

**Affiliations:** 1 Laboratory of Malaria and Vector Research, National Institute of Allergy and Infectious Diseases, National Institutes of Health, Bethesda, MD 20892, USA; 2 Rocky Mountain Laboratories, National Institute of Allergy and Infectious Diseases, National Institutes of Health, Hamilton, MT 59840, USA; 3 Laboratory of Proteomics and Analytical Technologies, SAIC-Frederick, Inc., National Cancer Institute, P.O. Box B, Frederick, MD 21702, USA

## Abstract

**Background:**

The salivary glands of hematophagous animals contain a complex cocktail that interferes with the host hemostasis and inflammation pathways, thus increasing feeding success. Fleas represent a relatively recent group of insects that evolved hematophagy independently of other insect orders.

**Results:**

Analysis of the salivary transcriptome of the flea *Xenopsylla cheopis*, the vector of human plague, indicates that gene duplication events have led to a large expansion of a family of acidic phosphatases that are probably inactive, and to the expansion of the FS family of peptides that are unique to fleas. Several other unique polypeptides were also uncovered. Additionally, an apyrase-coding transcript of the CD39 family appears as the candidate for the salivary nucleotide hydrolysing activity in *X.cheopis*, the first time this family of proteins is found in any arthropod salivary transcriptome.

**Conclusion:**

Analysis of the salivary transcriptome of the flea *X. cheopis *revealed the unique pathways taken in the evolution of the salivary cocktail of fleas. Gene duplication events appear as an important driving force in the creation of salivary cocktails of blood feeding arthropods, as was observed with ticks and mosquitoes. Only five other flea salivary sequences exist at this time at NCBI, all from the cat flea *C. felis*. This work accordingly represents the only relatively extensive sialome description of any flea species. Sialotranscriptomes of additional flea genera will reveal the extent that these novel polypeptide families are common throughout the Siphonaptera.

## Background

The blood-feeding habit evolved independently in many arthropod orders or even within insect families [1]. Although most orders containing hematophagous insects already existed in the Carboniferous period, fleas probably arose in the early Cretaceous, about 120 million years ago [2], but are thought to have expanded relatively recently, in the past 50 million years, where they spread simultaneously with mammals and birds [3]. For comparison, the Culicidae provided a common ancestor for anopheline and culicine mosquitoes over 150 million years ago [4], or 100 million years before the spread of fleas.

In their peculiar adaptation to blood feeding, arthropods had to prevent hemostasis and inflammatory mechanisms in the host, because these could disrupt the flow of blood or alert the host defense behavior. Therefore, arthropods evolved a salivary cocktail that prevents blood clotting and platelet aggregation, induces vasodilatation, and additionally modulates the immune and inflammatory responses. A large diversity of salivary pharmacologically active molecules is produced by both unrelated and related hematophagous insects. For example, in the Hemiptera, the salivary vasodilatation induced by the bed bug *Cimex lectularius *and kissing bug *Rhodnius prolixus *are both a result of nitric oxide, but they are carried out by completely different NO-carrying molecules; a lipocalin in the case of Rhodnius [5,6], and a modified inositol phosphatase in the case of Cimex [7,8]. In Diptera, the salivary anticlotting mechanisms also differ between anophelines and culicines, where a unique antithrombin molecule named anophelin is found in the Anopheles [9,10], and a serpin inhibitor of factor Xa occurs in *Aedes aegypti *[11]. The enzyme apyrase (ATP-diphosphohydrolase, catalysing the hydrolysis of both ATP and ADP to AMP) is ubiquitously found in the saliva of bloodsucking arthropods, where it destroys the inducer of platelet aggregation, ADP and the proinflammatory mediator ATP, released by damaged cells [12]; however, two very different protein families have been recruited to serve this function in insect saliva. In mosquitoes [13,14] and kissing bugs [15,16], 5' nucleotidase family members serve this function, while calcium-dependent Cimex apyrase family members are found in the bed bug [17] and sand flies [18]. It is clear that a convergent evolutionary scenario accounts for the diversity of pharmacologically active salivary components of hematophagous insects, generating a large diversity of novel compounds.

Four species of fleas are known to have salivary apyrase activity, but their enzymatic protein family is unknown [19,20], while *Xenopsylla cheopis *(Rots) and *Xenopsylla astia *were shown to have salivary anticlotting activity [21]. Hydrolysis of the lipidic agonist platelet activating factor (PAF) was also reported for the cat flea, but its molecular nature is unknown [22]. Other studies on flea saliva emphasize its allergenic role in human and veterinary medicine [23,24], including the molecular characterization of several antigens [25,26]. The paucity of salivary molecular information in fleas is indicated by the presence of only five proteins deposited in GenBank belonging to Siphonaptera and having the keyword 'salivary' (as in December 6, 2006), all belonging to the cat flea *Ctenocephalides felis*.

Despite great progress in the last 20 years in the identification of salivary pharmacologically active compounds, recent salivary transcriptome analysis of different bloodsucking arthropods is revealing a large number of putative secreted polypeptides for which the modes of action are unknown, including many novel protein families, some of which appear to be genus specific [27,28]. In the mosquitoes *Anopheles gambiae *and *Ae.aegypti*, over 70 salivary proteins have been identified; however, the function of fewer than 20 of these has been identified [28,29]. Given the phylogenetic distance between fleas and other bloodsucking arthropods, this work attempts to fill a gap in the knowledge of the salivary repertoire of hematophagous animals by providing insight into the sialome of the human vector of plague, *X.cheopis*. In this work, several transcripts coding for novel protein and peptide families are described and their translation into polypeptides confirmed. This work expands our knowledge in the salivary diversity of bloodsucking animals and, at the same time, it increases and maps our ignorance on the potential pharmacologic role of these novel protein families. To the extent that immune responses to flea saliva can control plague or rickettsial diseases for which fleas are vectors–as is the case of some other arthropodborne diseases [30-32]–this study also provides a platform of potential candidate antigens for future studies.

## Results and discussion

### cDNA library characteristics

A total of 944 clones were sequenced and used to assemble a database [see additional file 1] that yielded 245 clusters of related sequences, 171 of which contained only one expressed sequence tag (EST). The consensus sequence of each cluster is named either a contig (deriving from two or more sequences) or a singleton (deriving from a single sequence). For simplicity sake, this paper uses 'cluster' to denote sequences deriving both from consensus sequences and from singletons. The 245 clusters were compared using the program BlastX, BlastN, or RPSBLAST [33] to the nonredundant protein database of the National Center of Biological Information (NCBI), to a gene ontology database [34], to the conserved domains database of the NCBI [35], and to a customprepared subset of the NCBI nucleotide database containing either mitochondrial or rRNA sequences.

Because the libraries used are unidirectional, the three-frame translations of the dataset were also derived, and open reading frames (ORF) starting with a methionine and longer than 40 amino acid (AA) residues were submitted to SignalP server [36] to help identify putative secreted proteins. The EST assembly, BLAST, and signal peptide results were loaded into an Excel spreadsheet for manual annotation.

Four categories of expressed genes derived from the manual annotation of the contigs (Table 1). The putatively secreted (S) category contained 34% of the clusters and 75% of the sequences, with an average number of 8.4 sequences per cluster. The housekeeping (H) category had 43% and 17% of the clusters and sequences, respectively, and an average of 1.5 sequences per cluster. Twenty two percent of the clusters, containing 8.4% of all sequences, were classified as unknown (U), because no functional assignment could be made. Similar to the Hgroup, this category also had an average of 1.5 sequences per cluster. A good proportion of these transcripts could derive from 3^/ ^or 5^/ ^untranslated regions of genes of the above two categories, as was recently indicated for a sialotranscriptome of *An.gambiae *[29]. A possible transposable element originated 21 singletons representing either active transposition or, more likely, expression of transposable element regulatory transcripts in *X.cheopis*.

### Housekeeping (H) genes

The 106 clusters (comprising 157 EST) attributed to Hgenes expressed in the salivary glands of *X. cheopis *were further characterized into 13 subgroups according to function (Table 2). Not surprisingly for an organ specialized in secreting polypeptides, the two larger sets were associated with protein synthesis machinery (82 EST in 44 clusters) and energy metabolism (15 clusters containing 19 EST), a pattern also observed in other sialotranscriptomes [37-39]. We have also included in the H category a group of 12 EST that grouped into eight clusters, representing conserved proteins of unknown function, presumably associated with cellular metabolism. The fourth most abundant group, attributed to the protein modification function, is of interest due to the specialized function of the salivary glands. This group, comprising five clusters, includes a chaperone protein and enzymes that are associated with disulfide bridge formation. Several transporters were also identified, including those coding for two subunits of the VATPase complex, which has been shown to be necessary for mosquito salivation [40,41]. Transcripts coding for VATPases are a common finding in mosquito sialomes [37,39,42-44]. The complete list of all 106 gene clusters, along with further information about each, is given in additional file 2.

### Possibly secreted (S) class of expressed genes

Inspection of additional file 1 indicates the expression of several expanded gene families, including a family encoding proteins producing a match to KOG3720, indicative of lysosomal and prostatic acid phosphatases. This family alone is responsible for 35 clusters comprised of 268 sequences, or over 25% of the salivary library. Mucins are also represented, as well as an expansion of a peptide family unique to fleas that is related to the cat flea antigen annotated as FSH precursor (gi¦1575479). Several other novel peptide families, described in greater detail below, were also observed.

### Preliminary characterisation of the salivary proteome of *X. cheopis*

**Figure 1:**
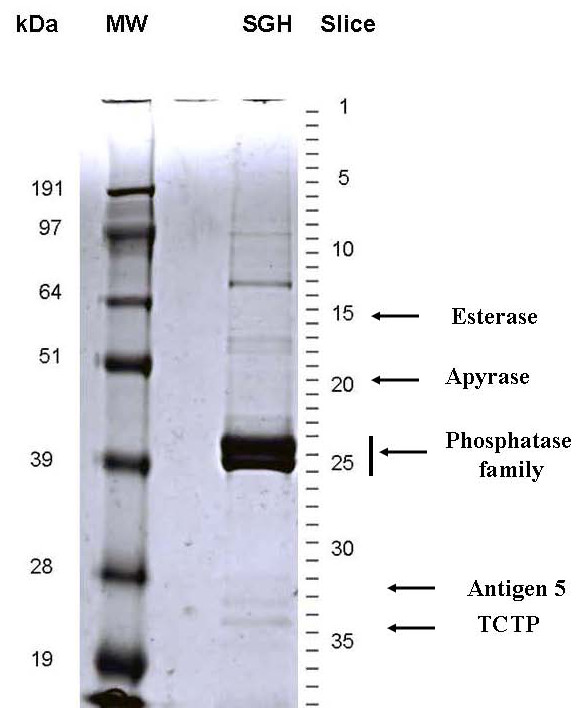
**Gel electrophoresis of *Xenopsylla cheopis *salivary gland homogenates**. The left portion of the gel shows molecular weight markers (MW) in kDaltons. The right portion shows the electrochromatogram of salivary gland homogenates, and the slices that were cut for tryptic digestion MS/MS experiments. The arrows point to the gel slices where the indicated enzymes and translationally controlled tumor protein (TCTP) were found. For more detail, see text.

To obtain information on protein expression in the salivary glands of *X. cheopis*, salivary gland homogenates (SGH) of approximately 100 pairs of glands were separated by SDS-PAGE. The stained protein bands were excised from the gel and submitted to tryptic digestion followed by MS/MS identification. Note that the electropherogram is dominated by two broad bands in the molecular mass range of 40–43 kDa (Figure 1). Only five slices of the gel shown in Figure 1 yielded useful information by being assigned to predicted sequences from our clusterized database and to fulllength sequences disclosed in this work. These results are summarized in Figure 1. To explore the expression of lower molecular mass polypeptides that were not represented in the SDS-PAGE experiment, we filtered 51 μg of homogenized salivary glands through a 30-kDa cutoff filter and submitted this filtrate to direct LC-MS/MS analysis. In addition, an aliquot of this filtrate was digested with trypsin prior to LC-MS/MS analysis. The low molecular weight (MW) filtrate yielded 22 additional matches with secreted proteins, as indicated in the supplemental Tables S1 and S2. The description of the identified proteins are embedded in the following manuscript sections.

### Analysis of the adult female *Xenopsylla cheopis* sialotranscriptome

Several clusters of sequences coding for housekeeping and putative secreted polypeptides indicated in additional file 1 are abundant and complete enough to extract consensus sequences of novel sequences. Additionally, we have performed primer extension studies in several clones to obtain full-or near full-length sequences of products of interest. A total of 76 novel sequences, 48 of which code for putative secreted proteins, are grouped together in additional file 2.

Following is a detailed description of the full-length transcripts found in the salivary glands of adult *X. cheopis:*

#### Putative salivary secreted proteins for which a protein family is known

##### Enzymes

###### Phosphatase family

Acid phosphatases catalyze the hydrolysis of phosphate monoesters and, in some cases, phosphoryl transfer between a phosphoester and alcohols [45]. These proteins are widely distributed in animal and plants, occurring in three different types. One type has a relatively small MW (18–20 kDa) and is found in mammalian liver, a second type has a higher MW (45–60 kDa), such as the enzymes of wheat germ, lysosomes and prostate. The third type are the purple acid phosphatases, which contain a binuclear iron center [46]. As listed in additional file 2, eight full-length transcripts coding for mature proteins ranging from 36–45 kDa all having a basic (> 8.5) pI were identified in this study. We also report two truncated (at the 5' end) members of this family. All transcripts of this class produce similar matches to lysosomal/prostatic acid phosphatases in the NR and GO databases, as well as to KOG3720 indicative of lysosomal and prostatic phosphatases. Except for one of the truncated members, several peptides were identified for each protein originating from the major band at 40 kDa reported in Figure 1. Edman degradation of the same band also revealed a strong signal for the proteins annotated as phos, phos1 and phos 1A.

**Figure 2:**
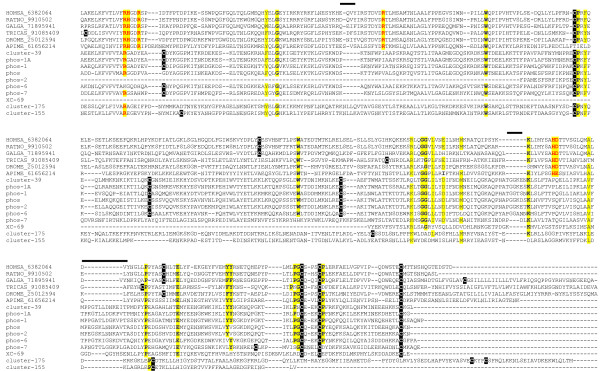
**Alignment of human, rat, chicken, beetle (*Triboleum castaneum)*, fly (*Drosophila melanogaster*), bee and flea acid phosphatase sequences**. The red letters over yellow background indicate amino acids found in the active center of rat and human enzymes. Other yellow background indicated identical (in bold) or conserved amino acids. The bars indicate regions of insertion/deletion when the flea and remaining sequences are compared. Cysteines are shown in white font over black background.

**Figure 3:**
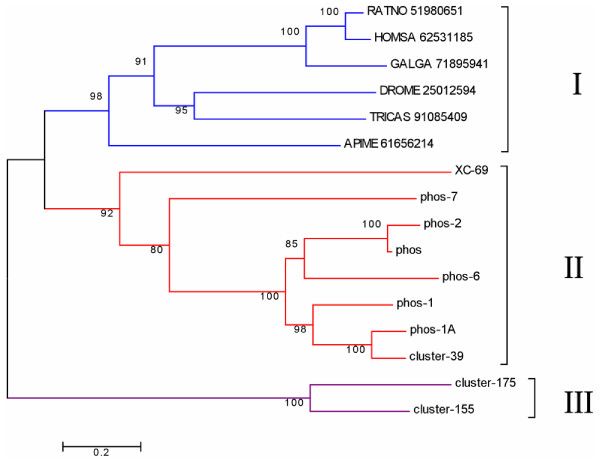
**Phylogram of human, rat, chicken, beetle (*Triboleum castaneum)*, fly (*Drosophila melanogaster*), bee and flea acid phosphatase sequences**. The phylogram was deducted from the Clustal alignment from figure 2. The non flea sequences are indicated by 5 letters representing the first 3 letters of the genus and 2 letters of the species name, followed by the NCBI accession number. The numbers in the phylogram indicates the percentage of concordance in 10,000 bootstraps. The bar at the bottom indicates 20% amino acid distance.

Alignment of the flea salivary phosphatase sequences with the vertebrates human, rat and chicken members, as well as with the venom phosphatase of the bee, a fly (*Drosophila melanogaster*) and a beetle (*Triboleum castaneum*) shows that, except for the first arginyl residue, all flea sequences do not have the conserved catalytic site residues as determined by crystal analysis of the human and rat sequences (shown in red color over yellow background in Figure 2) [46,47]. Remarkably absent is the histidyl residue located in the conserved motif RHGDR found in all non-flea sequences. This residue is known to be essential for catalysis, forming a phosphorylated intermediate during the reaction. All these active site residues experimentally determined for vertebrate enzymes are conserved in the fly, beetle and bee enzymes, indicating that fleas probably co-opted the acid phosphatase fold for a non-phosphatase function. Indeed, only a few other AA are conserved across the alignment shown in Figure 2, including the last three cysteines, a region of double aromatic AAs, and another region of double glycines, among others. Phylograms of the alignments show three distinct clades (Figure 3). Clade I has all non-flea enzymes, where three sub-families are found, the vertebrate subfamily (with six cysteines, four of which are involved in disulphide bonds) [46], the bee venom enzyme (with only three cysteines), and the fly and beetle enzymes (with five and seven cys residues, respectively). Clade II has eight of the ten flea sequences (with seven cysteines), and contains the flea sequences with the most conservation to the non-flea members, while clade III has the two most divergent flea sequences (with five and three cysteines). The odd number of cysteines in all invertebrate sequences indicate the possibility of reactive sulfhydryl groups in all enzymes, including those vertebrate enzymes where two of the six cysteines are not involved in disulphide bridges. It is apparent that fleas achieved a unique expansion of acid phosphatases, at least nine members of which were identified in the salivary glands using MS/MS. In contrast, only three genes are found for this class of enzymes in the *An. gambiae *proteome (by querying AnoXcel at the anobase site [48]) for the KOG motif lysosomal and prostatic acid phosphatases), or four such enzymes in the human genome [49].

The real substrate of acid prostatic/lysosomal phosphatases (where the enzyme can achieve 1 mg/ml in semen) [50] is not known, but it has been suggested that it can act as a protein tyrosine phosphate phosphatase and affect cell growth when the enzyme occurs intracellularly [51,52]. Extracellular protein phosphorylation/dephosphorylation is known to affect many aspects of cellular signaling [53,54], and is involved in platelet aggregation [55-57]. The conserved basic nature of all flea salivary phosphatases points to interaction with a negatively charged target. Although the active center of acid phosphatases appear quite open, accepting a diversity of small substrates, it is possible that the large size of the enzyme confers some degree of selectivity to the larger phosphorylated protein substrates. This selectivity is analogous to the large serine proteases that are involved in vertebrate blood clotting or invertebrate prophenoloxidase activation, which accepts many different small substrates but are quite specific for their protein substrates. Accordingly, it is possible that binding some host phosphorylated protein substrate has been the target of flea salivary phosphatases. Perhaps loss of the enzymatic activity kept the substrate permanently blocked by an inactive interaction, resulting in a more advantageous complete receptor blockage. On the other hand, loss of enzymatic function would require larger amounts of the protein to interact stoichiometrically with the target host protein, which might have been the reason for large protein expression and gene duplication observed, where gene duplication immediately confers the benefit of increased transcription. With time, gene duplicates may diverge to different targets, and/or to avoid immune detection by hosts. It is also possible that this phosphatase family may have evolved to chelate polyphosphates released by platelets that recently have been shown to have important hemostatic functions [58]. These considerations should help to identify the function of the flea salivary phosphatases.

###### Esterase

Additional file 2 reports two mRNA sequences coding for esterases, both similar to many insect proteins annotated as carboxylesterases. The XC-184 translation product of 211 aa is similar to the aminoterminal region of carboxylesterases with sizes varying between 530 and 560 aa [59]. The full polyadenylated transcript has clear polyadenylation sites and cannot transcribe for the larger homolog. Unless the EST is an artifact, it appears that flea saliva codes for a truncated version of a carboxylesterase enzyme. On the other hand, evidence for expression of the full-length esterase, consistent with a protein product of ~ 60 kDa, was obtained by the MS/MS experiments from gel slices 15 and 16 shown in Figure 1. Another esterase is coded by Cluster-136, a truncated mRNA coding for the carboxy terminus of a different carboxylesterase. Because we did not obtain the 5' region of this mRNA, we cannot verify whether the protein product has a signal peptide indicative of secretion. These mRNA sequences are included in the putative secreted category due to the possibility these enzymes may function as PAF hydrolases, as was found with an esterase salivary activity of the cat flea [22]. Esterases have been also reported in the salivary glands and saliva of both male and female adult *Ae. aegypti *mosquitoes [60], but their natural substrate is unknown.

###### Apyrase

**Figure 4:**
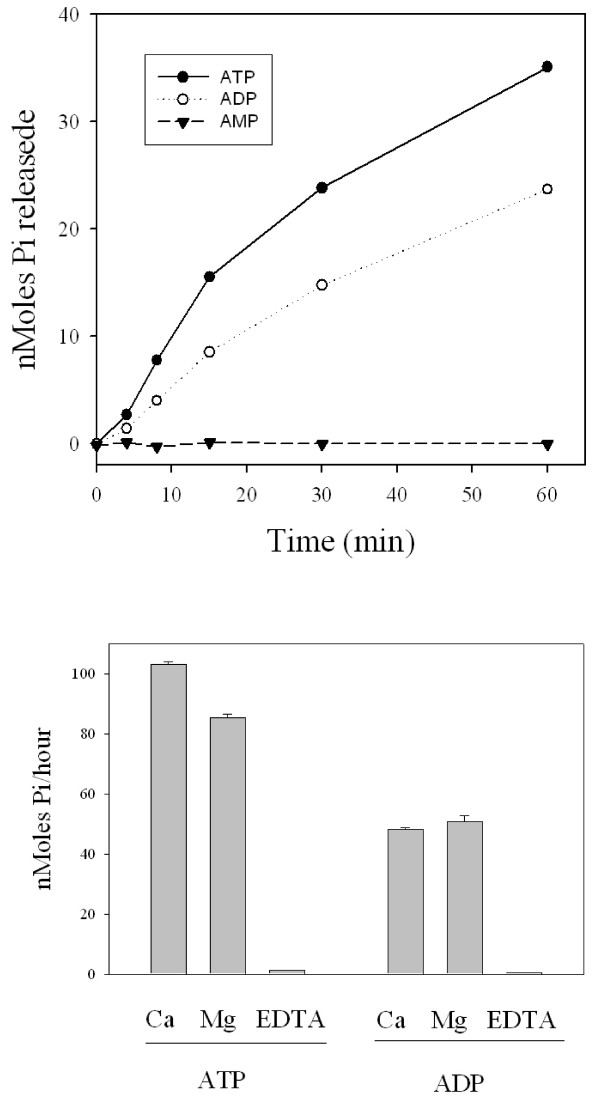
**Apyrase activity of salivary homogenates of the flea, *Xenopsylla cheopis***. Reaction media contained 50 mM TrisCl pH 7.4, 150 mM NaCl, 2 mM indicated nucleotide in 100 μl. The reaction started with addition of salivary gland homogenate to give 2.5 pairs per ml. (A) 1 mM CaCl_2 _plus 1 mM MgCl_2 _were added to the media before starting the reaction. (B) Either 2 mM CaCl_2_, 2 mM MgCl_2 _or 2 mM EDTA were added to the media before starting the reaction. The bars represent the average ± SE of 3 determinations. All incubations were done at 37°C.

**Figure 5:**
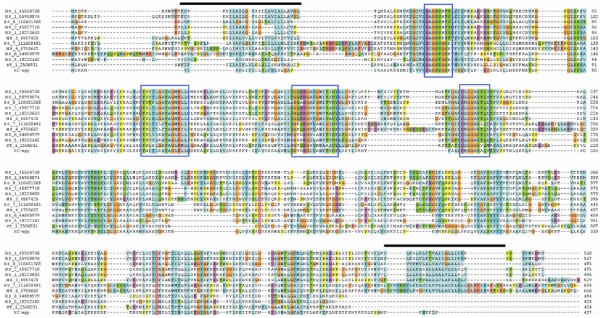
**Alignment of human, potato and flea proteins of the apyrase/CD39 family**. Human sequences are identified by HS_#_???? where # is the enzyme number and ???? refers to the NCBI gi accession number. The potato sequence is identified by ST_1 followed by the NCBI accession number. The flea sequence is indicated by the prefix XC. The boxes mark the 4 conserved domains identified by Wang et al [60]. The lines above the alignments indicate the regions of hydrophobic helices in the amino and terminal regions, the last of which is lacking in the four bottom sequences, representing the 2 human secreted enzymes, plus the potato and the flea enzymes.

Five transcripts coding for a homolog of an apyrase member of the CD39 family is the first finding of this type of gene expressed in the salivary glands of any hematophagous arthropod to date [Additional file 1]. The consensus sequences shown in additional file 1 indicates the EST are truncated and match the 3' region of homologous sequences, accounting for approximately 50% of the enzyme. This relatively abundant gene product may account for the salivary apyrase of this flea species. Because this activity was never described in *X. cheopis*, we investigated whether its SGH could hydrolyze adenosine nucleotides. Figure 4 indicates, as found for most salivary homogenates of bloodsucking arthropods, that *X.cheopis *has indeed a divalent cation dependent salivary apyrase activity, that can be activated by either Ca^++ ^or Mg^++^. This result is consistent with a CD39 or a 5'-nucleotidase family member, who functions with either divalent cation, but is not consistent with the Cimex apyrase type found in bed bugs and sand flies, which are strictly Ca^++ ^dependent. Protocols involving rapid amplification of cDNA ends (RACE) were used to obtain the 5' region of the truncated putative apyrase transcripts, yielding the full-length sequence coding for XC-APY, shown in additional file 2. When XC-APY was compared with eight members of the human CD39 family, plus the soluble potato apyrase sequence, the four conserved regions of the enzyme family are clearly found [61] (Figure 5 – boxed regions). Except for two human enzymes that are secreted, the remaining human sequences are membrane bound by two membrane anchors, one in each of the carboxy- or aminoterminal regions of the protein, indicated by the bars above the alignments in Figure 5, and by the predominance of aliphatic AA (shown in turquoise color in Figure 5). These hydrophobic regions coincide with membrane helices predicted by the TMHMM server, and can be seen in additional file 3[61]. These carboxyterminal helices are missing in the two secreted human enzymes [62,63], on the soluble potato apyrase, and in the flea apyrase (Figure 5). The flea apyrase also contains a clear signal peptide indicative of secretion, supporting the hypothesis that XC-apy is responsible for the salivary apyrase of *X.cheopis*. Mass spectrometry experiments located this enzyme within bands 19 and 20 of the gel shown in Figure 1, consistent with a MW of 45-50 kDa, and matching the predicted mass of the mature secreted enzyme of 46.9 kDa. To the extent that XC-apy is responsible for the observed salivary apyrase activity of *X.cheopis*, this will be the first description of a hematophagous arthropod to have co-opted this protein family for this particular activity.

###### Adenosine deaminase

Three transcripts code for truncated versions of the enzyme adenosine deaminase [Additional file 1], for which both transcripts and enzymatic activity were demonstrated in mosquito and sand fly salivary glands [64,65]. This salivary activity may help to convert mast cell degranulating adenosine into inosine [66].

##### Mucins

Three full-length transcripts with signal peptides indicative of secretion code for peptides of mature MW ranging from 5.5–13 kDa and having 6–12 putative galactosylation sites. They produce no similarity matches to any known proteins. Two of these products appear to result from a gene duplication event. Peptides with a high number of predicted galactosylation sites are regularly found in other hematophagous insect's sialotranscriptomes. These proteins may help to lubricate the insect food canal. Evidence for translation of XC-61 was found in bands 39 and 40 in the gel experiment of Figure 1, and also in the low MW filtrate. Expression of XC99 was indicated by a peptide match in the digested low MW filtrate.

##### Antimicrobial peptide

The full-length sequence for an antimicrobial peptide (AMP) of the defensin family is presented in additional file 2. Antimicrobial peptides are a regular finding in sialotranscriptomes of hematophagous insects and ticks. These peptides, when ingested with the blood meal, help control bacterial growth in the gut and may also protect their host-feeding lesions from infection.

##### Antigen 5 family

This protein family is widely found in the venom of vespids [67], in the salivary glands of many blood-sucking insects [37,44,68], and also as a multi-gene family in most animal genomes such as in *Drosophila *[69]. These proteins belong to the larger family of cysteine-rich extracellular proteins (CRISP) ubiquitously found in animals and plants [70]. Most proteins have no known function. We here report a salivary member of the antigen 5 family found in *X. cheopis*. Alignment and phylogenetic analysis of insect members of this family indicates, as expected, that *X. cheopis *salivary antigen 5 protein clusters with a related salivary protein from the cat flea (not shown).

#### Putative salivary secreted proteins belonging to novel polypeptide families

##### FS or antigen1 family

**Figure 6:**
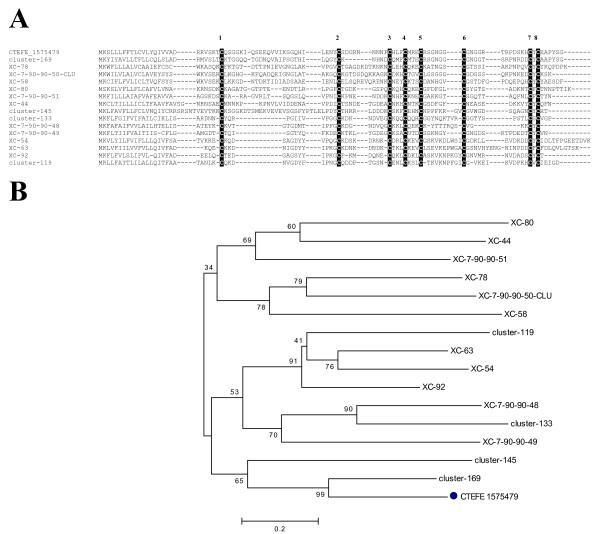
**The FS peptide family expressed in the salivary glands of *Xenopsylla cheopis***. A) Clustal aligment of the Oriental rat flea sequences with the cat flea sequence with NCBI accession number 1575479. Conserved cysteines are shown in reverse black color, and the number above their locations B) Phylogram of the FS family displaying the divergence of the family, and the association of the cat flea sequence with the rat flea sequence annotated as cluster-169. The numbers represent the percent concordance of 10,000 bootstrap replicates. The bar at the bottom indicates 20% amino acid distance.

The sialotranscriptome of *X. cheopis *revealed transcripts coding for several peptides with similarity to a previously described antigen of the cat flea named FS-H precursor, as well as two other cat flea larger proteins named FS-I and antigen 1 precursors. When the newly discovered peptide sequences were compared with the whole transcriptome database (using the tool BlastP with the low complexity filter off) other related sequences were found, thus creating a group of 15 related products that have in common solely the presence of eight cysteine residues (Figure 6A), the presence of signal peptides indicative of secretion, and a mature molecular mass varying from 6.3 to 9.2 kDa.

**Figure 7:**
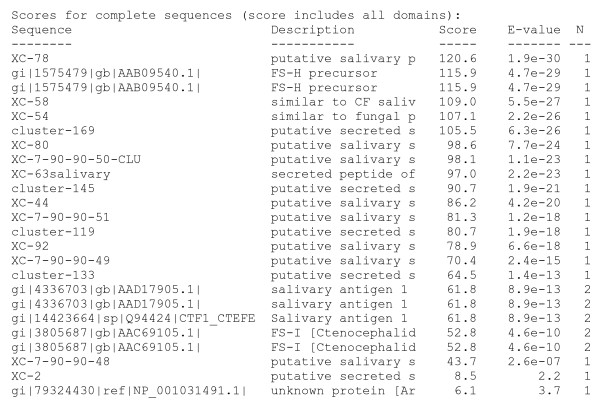
**Search of the non-redundant NCBI protein database for proteins similar to flea sequences found in this work**. A hidden Markov model was made from the alignment shown in Figure 6 (minus first 20 amino acids to exclude signal peptide) to search the non-redundant protein database.

The phylogram (Figure 6B) shows two unrelated groups of sequences, with strong bootstrap support for the relationship among several sequence pairs, including one pair consisting of one cat flea and one rat flea sequence. However, the roots of the several clades have weak bootstrap support indicating that these sequences either originated from unrelated genes, or more likely, they originated from gene duplication events with fast divergence from the ancestral gene. The programs hmmbuild and hmmcalibrate of the hmmer package (version 2.0) [71] were used to make a hidden Markov model from the alignment shown in Figure 6A (excluded of the first 20 AA containing most of the signal peptide region). The program hmmsearch from the same package was used to scan the NCBI nonredundant protein database with the flea sequences described here added. Only flea sequences were retrieved at a significant probability level (< 1e-7). Two additional sequences were retrieved at much lower significance levels, one being also a flea sequence, and one plant peptide (Figure 7). When the plant peptide was compared with the NR database (using BlastP) several defensinlike molecules from Arabidopsis and Helianthus were retrieved.

**Figure 8:**
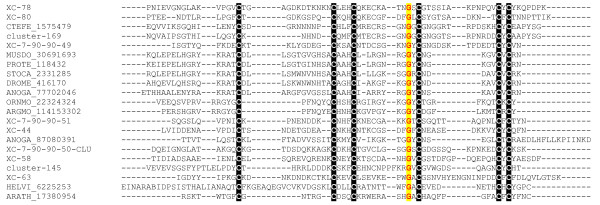
**Alignment of selected *Xenopsylla cheopis *peptides of the FS family with defensin peptides**. The conserved cysteine framework C...C-X-X-X-C...G-X-C ...C-X-C is indicated. Sequences from the present work start with XC or cluster; other sequences are from GenBank and consist of 5 letters and their gi¦ accession number. The five letters represent the genus name (first 3 letters) followed by 2 letters from the species name; Accordingly, CTEFE: *Ctenocephalides felix*, MUSDO: *Musca domestica*, PROTE: *Protophormia terraenovae*, STOCA: *Stomoxys calcitrans*, DROME: *Drosophila melanogaster*, ANOGA: *Anopheles gambiae*, ORNMO:*Ornithodorus moubata*, ARGMO: *Argas monolakensis*, HELVI:*Heliothis virescens*, ARATH: *Arabidopsis thaliana*. The aminoterminal part of the peptides is not shown.

Plant and insect defensins as well as the scorpion toxins show a high degree of structural conservation despite considerable divergence of sequence [72]. These peptides adopt a cysteine-stabilized-αβ fold containing a three stranded β-sheet and a single α-helix [72,73]. The cysteine sequence motif C...CXXXC...GXC...CXC is conserved in this group, where X is any amino acid and ... is a variable interval of AA [72]. The six cysteine residues contained in the motif form three disulfide bonds, and a fourth disulfide bond is often present, but its position is variable. Sequence alignments of FS family of flea salivary peptides with insect/plant defensins reveal only low overall identity. However, the three disulfide bond cysteine sequence motif characteristic of defensins and scorpion toxins is present (Figure 8), making it likely that these peptides belong to the cysteine stabilized αβ fold group. Insect type defensins are antimicrobial molecules important for protection against bacteria and fungi [74], while scorpion toxins are neurotoxic molecules that impair the functions of sodium and potassium channels [75]. Based on this similarity, possible functions of this group of peptides could include, but not be limited to, controlling microbial growth or analgesic function.

The digested or undigested 30 kDa filtered SGH provided for MS/MS identification of 11 of the 15 peptides reported in additional file 2, while the front of the gel shown in Figure 1 allowed for identification of two peptides, also identified by MS/MS of the filtrated sample (for details see Methods section).

##### Other novel peptides

Fifteen other polypeptides are described in additional file 2, all containing a signal peptide indicative of secretion, and without significant matches to other proteins in the NR database, except for the peptide named Cluster-149 that is weakly similar to low complexity bacterial proteins. Two of these peptides appear to be splice variants (XC-42 and XC-43) or products of recent gene duplication. Two other peptides (XC-3 and XC-56) share significant homology in their signal peptides, but not so much in their mature peptide sequences. Except for Cluster-149 that codes for a mature protein of 19.3 kDa, all other peptides have a predicted weight smaller than 7.8 kDa, six of which are less than five kDa. XC-105, coding for a mature peptide of 3.9 kDa has a weak SMART match to calcitonin or calcitonin gene related peptides, the last one being a potent vasodilator [76]. XC-56 is weakly similar to HIV gp160 and to patented peptides with inhibitory activity towards the viral protein binding to the CCR5 receptor. Cluster-113 peptide has a cysteine scaffold found in the cysteine rich region of tick salivary metalloproteases, suggestive of an interaction with extracellular matrix elements. XC-2 has sequence homology to patented decapeptides similar to peptide hormone receptors, and to small sequence regions of these receptors. XC-68 has the cysteine scaffold of arthropod defensins, including the C-X-C carboxyterminus where X is a hydrophobic AA (Ile in this case). Mass spectrometry experiments indicated in additional file 2 produced evidence for expression of 9 of the 15 polypeptides.

##### Housekeeping proteins

Additional file 2 describes 28 full-length sequences coding for 20 proteins involved in protein synthesis (mostly ribosomal proteins), two that are part of the proteasome machinery, one involved in protein modification, one cytoskeletal protein, one involved in energy metabolism and two that are uncharacterised, conserved proteins. These sequences can help in phylogenetic studies and as controls in future studies with *X.cheopis*.

## Conclusion

Analysis of the salivary transcriptome of the flea *X. cheopis *revealed the unique pathways taken in the evolution of the salivary cocktail of fleas. Gene duplication events led to large expansions of a family of probably inactive acidic phosphatases, never found before in any other blood-sucking arthropod, and to the FS family of peptides unique to fleas. Additionally, over a dozen unique peptides were found. An apyrase-coding transcript of the CD-39 family appears as the candidate for the salivary nucleotide hydrolyzing activity in *X. cheopis*. If this is confirmed, this will be the first arthropod to have recruited this gene family for its salivary apyrase activity. Only five other flea salivary sequences exist at this time at NCBI, all from the cat flea *C. felis*. This work accordingly represents the only relatively extensive sialome description of any flea species. Sialotranscriptomes of additional flea genera will reveal the extent that these novel polypeptide families are common throughout the Siphonaptera.

## Methods

### Fleas

Intact salivary gland pairs were collected from adult female *X. cheopis *fleas. Individual fleas (anesthetized by chilling on ice) were dissected in 10 μl of PBS on a glass microscope slide on the stage of a dissecting stereomicroscope. By grasping the dorsal half of the flea above the forelegs with forceps, pressing down on the abdomen just posterior to the midgut with a bent dissecting needle, and pulling, the two pairs of salivary glands and the midgut would usually remain attached to the head and be pulled free of the rest of the body. The common lateral salivary ducts were cut to release each pair, which were then hooked with a dissecting pin and placed in PBS at 4°C. Pools of 40 pairs of glands in 20 μl PBS were frozen at 70°C. Glands used for apyrase assays were dissected and stored in 10 mM TrisHCl and 150 mM NaCl, pH 7.4 rather than PBS.

### Salivary gland isolation and library construction

*X. cheopis salivary *gland mRNA was isolated from 200 salivary gland pairs from adult fleas using the Micro-FastTrack mRNA isolation kit (Invitrogen, SanDiego, CA). The PCR-based cDNA library was made following the instructions for the SMART cDNA library construction kit (Clontech, Palo Alto, CA). This system utilizes oligoribonucleotide (SMART IV) to attach an identical sequence at the 5' end of each reverse-transcribed cDNA strand. This sequence is then utilized in subsequent PCR reactions and restriction digests.

First strand synthesis was carried out using PowerScript reverse transcriptase at 42°C for 1 hr in the presence of the SMART IV and CDS III (3') primers. Second strand synthesis was performed by a long distance (LD) PCR-based protocol, using Advantage™ Taq Polymerase (Clontech) mix in the presence of the 5' PCR primer and the CDS III (3') primer. The cDNA synthesis procedure resulted in the creation of *SfiI A *&*B *restriction enzyme sites at the ends of the PCR products that are used for cloning into the phage vector. The PCR conditions were: 95°C for 20 sec; 24 cycles of 95°C for 5 sec., 68°C for 6 min. A small portion of the cDNA obtained by PCR was analysed on a 1.1% agarose gel to check for the quality and range of cDNA synthesised. Double stranded cDNA was immediately treated with proteinase K (0.8 μg/ml) at 45°C for 20 min and the enzyme was removed by ultrafiltration though a Microcon (Amicon) YM100 centrifugal filter device. The cleaned, doublestranded cDNA was then digested with *SfiI *at 50°C for 2 hrs, followed by size fractionation on a ChromaSpin-400 column (Clontech, Palo Alto, CA). The profile of the fractions was checked on a 1.1% agarose gel and fractions containing cDNAs of more than 400 bp were pooled and concentrated using a Microcon YM100.

The cDNA mixture was ligated into the λ TriplEx2 vector (Clontech, Palo Alto, CA) and the resulting ligation mixture was packaged using the GigaPack^® ^III Plus packaging extract (Stratagene, La Jolla, CA) according to the manufacturer's instructions. The packaged library was plated by infecting log phase XL1 Blue *E. coli *cells (Clontech, Palo Alto, CA). The percentage of recombinant clones was determined by performing a blue-white selection screening on LB/MgSO_4 _plates containing X-gal/IPTG. Recombinants were also determined by PCR, using vector primers (5' λ TriplEx2 Sequencing Primer and 3' λ TriplEx2 Sequencing) flanking the inserted cDNA and visualising the products on a 1.1% agarose/EtBr gel.

### Sequencing of the *X. cheopis* cDNA library

The *X. cheopis *salivary gland cDNA library was plated on LB/MgSO_4 _plates containing X-gal/IPTG, to an average of 250 plaques per 150 mm Petri plate. Recombinant (white) plaques were randomly selected and transferred to 96-well MICROTEST™ U Bottom plates (BD BioSciences, Franklin Lakes, NJ), containing 100 μl of SM buffer [0.1 M NaCl; 0.01 M MgSO_4; _7H_2 _O; 0.035 M TrisHCl (pH 7.5); 0.01% gelatin] per well. The plates were covered and placed on a gyrating shaker for 30 min at room temperature. The phage suspension was either immediately used for PCR or stored at 4°C for future use.

To amplify the cDNA using a PCR reaction, four microliters of the phage sample was used as a template. The primers were sequences from the λ TriplEx2 vector and named pTEx2 5 seq (5'TCC GAG ATC TGG ACG AGC 3') and pTEx2 3 LD (5' ATA CGA CTC ACT ATA GGG CGA ATT GGC 3'), positioned at the 5' end and the 3' end of the cDNA insert, respectively. The reaction was carried out in 96 well flexible PCR plates (Fisher Scientific, Pittsburgh, PA) using the TaKaRa EX Taq polymerase (TAKARA Mirus Bio, Madison, WI), on a Perkin Elmer GeneAmp^® ^PCR system 9700 (Perkin Elmer Corp., Foster City, CA). The PCR conditions were: one hold of 95°C for 3 min; 25 cycles of 95°C for 1 min, 61°C for 30 sec; 72°C for 2 min. The amplified products were analysed on a 1.5% Agarose/EtBr gel. 1100 cDNA library clones were PCR amplified and the ones showing single band were selected for sequencing. Approximately 200–250 ng of each PCR product was transferred to Thermo-Fast 96-well PCR plates (ABgene Corp., Epsom, Surray, UK) and frozen at 20°C, before cycle sequencing using an ABI3730 XL machine.

### Bioinformatic tools and procedures used

Expressed sequence tags (EST) were trimmed of primer and vector sequences, clusterized, and compared with other databases as described [44]. The BLAST tool [77], CAP3 assembler [78], ClustalW [79], and Treeview software [80] were used to compare, assemble, and align sequences and to visualise alignments. For functional annotation of the transcripts we used the tool BlastX [33] to compare the nucleotide sequences to the NR protein database of the National Center for Biotechnology Information (NCBI) and to the Gene Ontology (GO) database[34]. The tool RPSBlast [33] was used to search for conserved protein domains in the Pfam [81], SMART [82], Kog [83] and Conserved Domains Databases (CDD) [35]. We have also compared the transcripts with other subsets of mitochondrial and rRNA nucleotide sequences downloaded from NCBI, and to several organism proteomes downloaded from NCBI (yeast), Flybase (*Drosophila melanogaster*), or ENSEMBL (*An. gambiae*). Segments of the three-frame translations of the EST (because the libraries were unidirectional we did not use six-frame translations), starting with a methionine found in the first 100 predicted AA, or to the predicted protein translation in the case of complete coding sequences, were submitted to the SignalP server [36] to help identify translation products that could be secreted. O-glycosylation sites on the proteins were predicted with the program NetOGlyc ([84]. Functional annotation of the transcripts was based on all the comparisons above. Following inspection of all these results, transcripts were classified as either Secretory (S), Housekeeping (H) or of Unknown (U) function, with further subdivisions based on function and/or protein families. Phylogenetic analysis and statistical Neighbor Joining (NJ) bootstrap tests of the phylogenies were done with the Mega package [85].

### Gel electrophoresis studies

Flea salivary proteins representing approximately 100 gland pairs were resolved by one-dimensional (1D) sodium dodecylsulfate polyacrylamide gel electrophoresis (4–12% gradient gels) and visualised with Coomassie blue staining (Pierce, Rockford, IL). Excised gel bands were destained using 50% acetonitrile in 25 mM NH_4_HCO_3_, pH 8.4 and vacuum dried. Trypsin (20 μg/mL in 25 mM NH_4_HCO_3_, pH 8.4) was added and the mixture was incubated on ice for one hr. The supernatant was removed and the gel bands were covered with 25 mM NH_4_HCO_3_, pH 8.4. After overnight incubation at 37°C, the tryptic peptides were extracted using 70% acetonitrile, 5% formic acid, and the peptide solution was lyophilised and desalted using ZipTips (Millipore, Bedford, MA).

### Low MW fractionation of flea salivary proteins

A low molecular protein sample was prepared by resuspending 51 μg of total flea protein salivary homogenate into 2 mL of 100 mM NH_4_HCO_3_, pH 8.4, containing 10% acetonitrile. Low MW proteins were obtained by centrifugal ultrafiltration using Centriplus 30 kDa ultrafilters (Millipore, Billerica, MA) spun at 750 × *g *at 4°C. The low MW filtrate was lyophilised and resuspended in 50 μL of 25 mM NH_4_HCO_3_, pH 8.4, and half of the solution was digested with trypsin (enzyme:protein ratio of 1:50) for 16 h at 37°C. The undigested and digested samples were desalted using C18 ZipTips (Millipore, Bedford, MA), lyophilised to dryness and resuspended in 14 μL 0.1% TFA for subsequent nanoRPLCMS/MS analysis.

### Nanoflow reversedphase liquid chromatography tandem mass spectrometry (nanoRPLCMS/MS)

The tryptic peptides were analyzed using nanoRPLCMS/MS. A 75 μm i.d. × 360 μm o.d. × 10 cm long fused silica capillary column (Polymicro Technologies, Phoenix, AZ) was packed with 3 μm, 300 Å pore size C-18 silica bonded stationary RP particles (Vydac, Hysperia, CA). The column was connected to an Agilent 1100 nanoLC system (Agilent Technologies, Palo Alto, CA) that was coupled online with a linear iontrap (LIT) mass spectrometer (LTQ, ThermoElectron, San Jose, CA). The peptides were separated using a gradient consisting of mobile phase A (0.1% formic acid in water) and B was (0.1% formic acid in acetonitrile). The peptide samples were injected and gradient elution was performed under the following conditions: 2% B at 500 nL/min in 30 min; a linear increase of 2–42% B at 250 nL/min in 110 min; 42–98% in 30 min including the first 15 min at 250 nL/min and then 15 min at 500 nL/min; 98% at 500 nL/min for 10 min. The LIT-MS was operated in a datadependent tandem MS (MS/MS) mode in which the five most abundant peptide molecular ions in every MS scan were selected for collision induced dissociation (CID) using a normalized collision energy of 35%. Dynamic exclusion was applied to minimize repeated selection of previously analyzed peptides. The capillary temperature and electrospray voltage were set to 160°C and 1.5 kV, respectively. Tandem MS spectra from the nanoRPLCMS/MS analyses were searched against a protein fasta database derived from the flea salivary gland, using SEQUEST operating on an 18 node Beowulf cluster. For a peptide to be considered legitimately identified, it had to achieve stringent charge state and proteolytic cleavage-dependent cross correlation (X_corr_) and a minimum correlation (ΔC_n_) score of 0.08.

### Measurement of apyrase activity

Apyrase activity was measured as described previously [86]. Specific conditions are given in the legend accompanying Figure 4.

## Authors' contributions

JFA made cDNA library, supervised DNA sequencing and RACE extensions, contributed to work supervision, data analysis and manuscript writing. BJH contributed salivary glands and manuscript writing.

DAL contributed to mass spectrometry part of the work and manuscript writing. TPC contributed to mass spectrometry part of the work and manuscript writing. TDV contributed to mass spectrometry part of the work and manuscript writing. VMP performed cloning and sequencing of the cDNA library. JMCR contributed to work supervision, apyrase experiments, data analysis and manuscript writing. All authors read and approved the final manuscript.

## Supplementary Material

Additional file 1 **Supplemental table S1**. Hyperlinked Microsoft Excel file with assembled EST's and various database comparisons. This file can be found at NCBI [87] and at BMC Genomics as supplemental data. Click here for file

Additional file 2 **Supplemental table S2**. Hyperlinked Microsoft Excel file with predicted salivary proteins and various database comparisons. This file can be found at NCBI [88] and at BMC Genomics as supplemental data. Click here for file

Additional file 3 **Supplemental table S1**. Hyperlinked Microsoft Excel file with CD39/apyrase proteins and various database comparisons. This file can be found at NCBI [89] and at BMC Genomics as supplemental data. Click here for file

## Figures and Tables

**Table 1:** Transcript abundance according to functional class

**Class**	Clusters *	**Sequences**	**Sequences/Cluster**
Housekeeping	106 (43.3)	157 (16.6)	1.48
Secreted	84 (34.3)	707 (74.9)	8.42
TE	1 (0.4)	1 (0.1)	1.00
Unknown	54 (22.0)	79 (8,4)	1.46
			
Total	245	944	

* Number (Percent of total)

**Table 2:** Functional classification of housekeeping transcripts

**Function**	**Clusters**	**Sequences**	**Sequences/Cluster**
Protein synthesis	53	82	1.55
Energy metabolism	15	19	1.27
Unknown conserved	8	12	1.50
Protein modification	5	6	1.20
Proteasome machinery	5	7	1.40
Protein export	4	4	1.00
Transcription machinery	4	9	2.25
Transporters/storagers	4	4	1.00
Signal transduction	3	3	1.00
Cytoskeletal	2	8	4.00
Cuticle protein	1	1	1.00
Nuclear regulation	1	1	1.00
Oxidative metabolism	1	1	1.00

Total	106	157	
